# Caspases promote cell proliferation after necrosis

**DOI:** 10.7554/eLife.103786

**Published:** 2024-11-04

**Authors:** Prathibha Yarikipati, Andreas Bergmann

**Affiliations:** 1 Department of Molecular, Cell and Cancer Biology, UMass Chan Medical School Worcester United States

**Keywords:** necrosis, apoptosis, regeneration, cell death, caspases, *D. melanogaster*

## Abstract

An enzyme known as caspase, which initiates apoptosis, has a central role in the regeneration of cells and repair of tissue that can occur after necrosis.

**Related research article** Klemm JW, Van Hazel C, Harris RE. 2024. Regeneration following tissue necrosis is mediated by non-apoptotic caspase activity. *eLife* **13**:RP101114. doi: 10.7554/eLife.101114.

Necrosis is a form of cell death that can be caused by illness, infection, injury, radiation or chemicals. During necrosis, cells swell, burst and lyse, spilling their contents into the extracellular space, which attracts cells of the immune system and triggers inflammation. In humans, necrosis is irreversible and in severe cases, can lead to systemic organ failure and death (Khalid and Azimpouran, 2023).

However, recent research on fruit flies has revealed that some necrotic tissue can have surprisingly regenerative capabilities (Klemm et al., 2021; Yang et al., 2013). In these studies, necrosis was genetically induced in the wing imaginal disc, the precursor of the adult wings, by expressing a mutant Glutamate receptor (GluR1) that triggers the influx of calcium ions into cells, causing them to burst and die. Over half of these flies had wings that appeared normal, suggesting that the necrotic damage caused by GluR1 must have been repaired or regenerated during wing development.

Apoptosis is a genetically controlled form of cell death mediated by enzymes called caspases, and GluR1-induced necrosis is associated with two batches of apoptotic cells. The first batch is found at the edge of the wound, while the second batch, termed necrosis-induced apoptotic cells, is found away from the wound. It is likely that the role of the first batch is to clear away cells from the site of the wound. Now, in eLife, Jacob Klemm, Chloe Van Hazel and Robin Harris at the Arizona State University report on the properties of the cells in the second batch (Klemm et al., 2024).

The researchers used the same genetic ablation system (expression of a leaking GluR1 channel) to induce necrotic cell death within the wing imaginal disc of fruit flies. This revealed that inhibition of apoptosis in the second batch of cells significantly inhibited the regenerative proliferation of necrotic tissue. This suggests that controlled apoptosis is necessary for effective tissue regeneration after necrosis. However, necrosis-induced apoptosis occurs shortly after the induction of necrosis, while regenerative proliferation only occurs much later (36–48 hours; Figure 1A–C). How can necrosis-induced apoptotic cells induce regenerative proliferation if they have already died?

**Figure 1.**
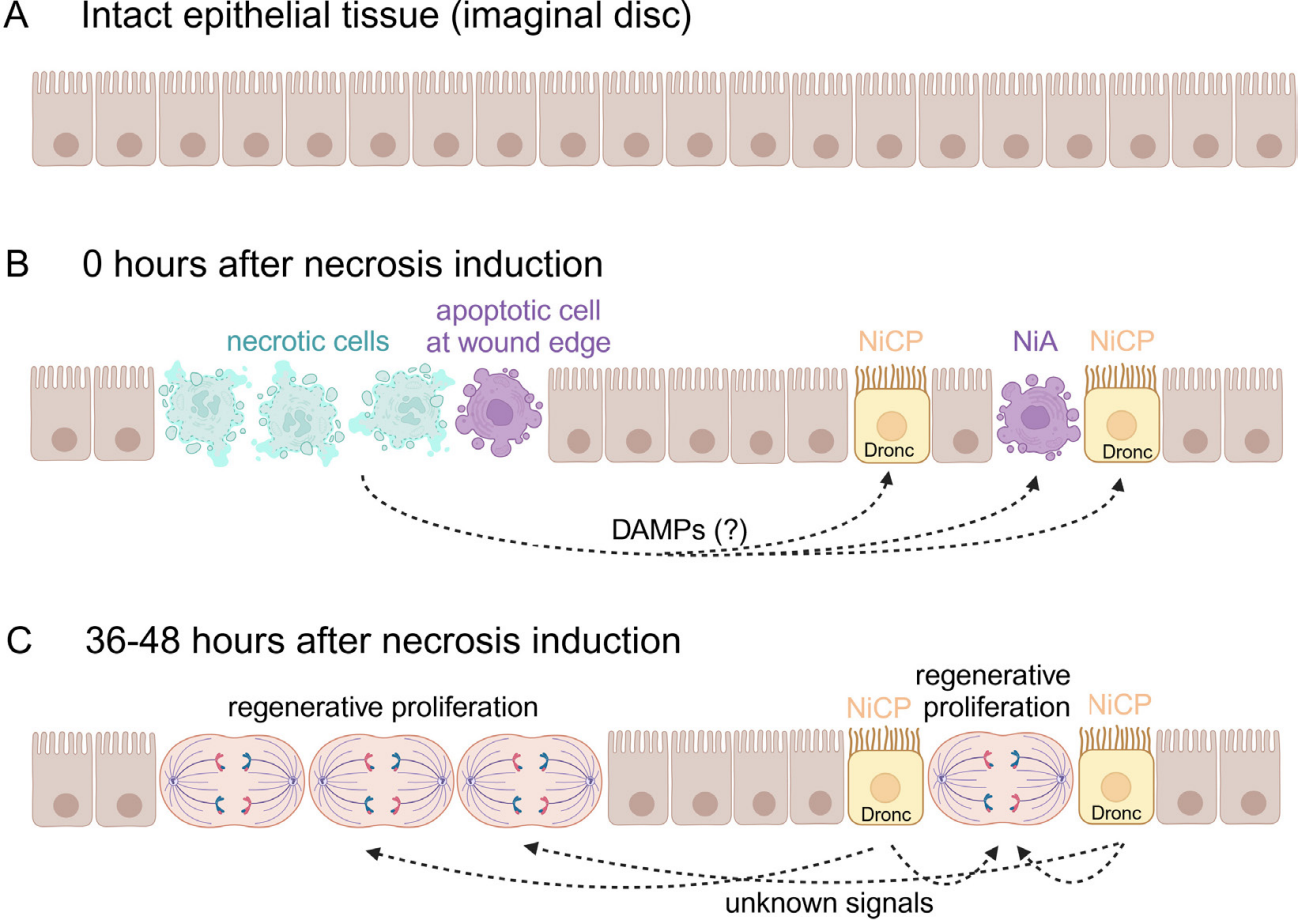
Regenerative proliferation and tissue repair after induction of necrosis. (**A**) The wing imaginal disc is composed of a monolayer of epithelial cells. (**B**) Klemm et al. genetically induced necrosis. Immediately afterwards (0 hours), necrotic cells (pale green) and two batches of apoptotic cells (purple) were detectable. The first batch of apoptotic cells was at the edge of the wound, next to the necrotic cells. The second batch was located away from the wound and consisted of necrosis-induced apoptotic (NiA) cells, which died, and necrosis-induced caspase-positive (NiCP) cells, which did not die despite the fact that they contained the caspase Dronc, which can initiate apoptosis. (**C**) After 36–48 hours, the necrotic and apoptotic cells have been removed. However, the NiCP cells persist and release unknown signals in a Dronc-dependent manner that triggers regenerative proliferation and promotes the repair of the necrotic wound. This figure was created with BioRender.com.

Klemm et al. found that a significant fraction of necrosis-induced apoptotic cells did not undergo apoptosis. Instead, these cells – called necrosis-induced caspase positive (NiCP) cells because they contain caspases – survived and promoted regenerative proliferation. The experiments revealed that the regenerative proliferation mediated by NiCP cells depends on Dronc, a caspase that initiates apoptosis, but not on the effector caspases that execute apoptosis. Even though the effector caspases were active, they were only present at sub-lethal levels, which would explain why the NiCP cells survived during the regenerative process.

Dronc also has non-apoptotic functions. Indeed, it can mediate the proliferation of nearby cells in a process called apoptosis-induced proliferation, in which apoptotic cells can influence neighbouring cells to proliferate (Fogarty and Bergmann, 2017). Dronc’s function in regenerative proliferation after necrosis appears to be distinct from the mechanisms by which apoptosis might drive cell division, suggesting that Dronc can control regenerative proliferation through at least two non-apoptotic mechanisms: through activating signalling pathways that promote cell growth in apoptosis-induced proliferation, and through regulating cell division in NiCP-mediated regeneration (Figure 1C). These results suggest a versatile role for Dronc in proliferation and tissue regeneration beyond its canonical function in apoptosis.

The findings by Klemm et al. challenge the traditional view of apoptosis as being a solely destructive process and highlight its potential role in tissue repair and regeneration. They also underscore the interplay between different forms of cell death (necrosis and apoptosis) in tissue homeostasis and repair.

However, a number of questions about the complex interplay between cell death and regeneration remain open. For example, what signals mediate the formation of necrosis-induced apoptotic cells and NiCP cells away from the wound, and what receptors do these signals target? Possible candidates could be intracellular factors, called DAMPs (damage-associated molecular patterns), which are released during the lysis of necrotic cells. Moreover, we need to understand the non-apoptotic roles of Dronc more fully, and to establish how NiCP cells maintain effector caspases below a sub-lethal threshold to stay alive. It would also be interesting to see what role macrophages and other innate immune cells play in the regenerative response to necrosis.

Lastly, although necrotic damage can be induced in the entire wing disc, only cells in the developing wing pouch (and, to a much lesser extent, cells in the notum), are competent to become NiCPs and to induce regenerative proliferation, whereas cells in the hinge region of the disc are not. By understanding the factors that confer regenerative competence to specific regions of the wing disc, new fundamental principles of tissue regeneration may be uncovered. These insights could potentially be leveraged to develop novel therapeutic approaches for enhancing regenerative capacity in human tissues affected by necrotic damage, opening new avenues in regenerative medicine.
